# Wessex Rahere Club

**Published:** 1963-10

**Authors:** 


### WESSEX RAHERE CLUB

The October meeting will take place at the White Hart Hotel, Salisbury, on October 19th 1963, when Mr. Geoffrey Bourne will be the Guest of Honour.

Any Bart's graduates who have not received a notice, or who wish to obtain further information about the Club should get in touch with the Honorary Secretary, Mr. A. Daunt Bateman, at 11, The Circus, Bath.

